# A review: Music-emotion recognition and analysis based on EEG signals

**DOI:** 10.3389/fninf.2022.997282

**Published:** 2022-10-25

**Authors:** Xu Cui, Yongrong Wu, Jipeng Wu, Zhiyu You, Jianbing Xiahou, Menglin Ouyang

**Affiliations:** ^1^The Art School, Xiamen University, Xiamen, China; ^2^Department of Software Engineering, Xiamen University, Xiamen, China; ^3^Shenzhen Institutes of Advanced Technology, Chinese Academy of Sciences, Shenzhen, China; ^4^National Institute for Data Science in Health and Medicine, School of Medicine, Xiamen University, Xiamen, China; ^5^The Mathematics and Computer School, Quanzhou Normal University, Quanzhou, China; ^6^The Affiliated Hospital of Medical School, Ningbo University, Ningbo, China

**Keywords:** EEG, emotions, music, recognition, song

## Abstract

Music plays an essential role in human life and can act as an expression to evoke human emotions. The diversity of music makes the listener's experience of music appear diverse. Different music can induce various emotions, and the same theme can also generate other feelings related to the listener's current psychological state. Music emotion recognition (MER) has recently attracted widespread attention in academics and industry. With the development of brain science, MER has been widely used in different fields, e.g., recommendation systems, automatic music composing, psychotherapy, and music visualization. Especially with the rapid development of artificial intelligence, deep learning-based music emotion recognition is gradually becoming mainstream. Besides, electroencephalography (EEG) enables external devices to sense neurophysiological signals in the brain without surgery. This non-invasive brain-computer signal has been used to explore emotions. This paper surveys EEG music emotional analysis, involving the analysis process focused on the music emotion analysis method, e.g., data processing, emotion model, and feature extraction. Then, challenging problems and development trends of EEG-based music emotion recognition is proposed. Finally, the whole paper is summarized.

## 1. Introduction

Music is a product created by musicians in the brain and mapped to reality through playing and is also a meaningful way to express emotions and generate emotional resonance. Music is a sound symbol representing people's thoughts and has specific acoustic and structural characteristics. The structure and features of music, such as pitch, tonality, rhythm, and volume, play an essential role in emotional expression. For example, fast rhythms (dense rhythms) typically indicate high arousal levels, while slow rhythms (sparse rhythms) indicate low arousal emotions. Music has many elements, including loudness, pitch, beat, tempo, rhythm, melody, harmony, texture, style, timbre, dynamics, structure, and more. Generally speaking, music has three essential characteristics: loudness, pitch, and timbre (Lin et al., 2010). Music is a type of sound caused by objects vibrating the air regularly. It enters the brain through the human ear and acts on the cranial nerves. Besides, music is one of the ways of expressing emotions, which can evoke strong emotional responses from listeners with good cross-cultural, consistent consistency. Listening to music is an easy and effective way to change your mood or reduce stress. Neuroscientific research has found that the melody and rhythm of music directly affect the human ear, and the listener can perceive the emotion expressed by music in the subconscious state. Studies have shown that music can induce various emotions in humans, including sadness, nostalgia, tension, happiness, relaxation, calmness, and joy. Listening to music causes emotional mechanisms that are very complex, which often involves a series of related interdisciplinary knowledge such as psychology, physiology, and neuroscience. It isn't easy to find an intuitive quantitative method to analyze. When musical stimulation acts on a person's auditory system, the brain will temporarily increase the activity of specific nerve cells, thereby affecting the expression of people's emotions. Music signals and EEG are closely related in the root (Lin et al., 2006). The EEG often used to verify the influence of music on human brain activity. Studying the relationship between EEG signals and music can help discover how music activates corresponding neurons in the brain. And it can also affect the expression of human emotions. Therefore, studying the correlation between music and EEG signals is of great significance.

Human emotion is a complex phenomenon closely related to human mental state and emotion, including psychological, physiological, and social aspects (Cacioppo et al., 2021). Emotions can be described as changes in the form of the human brain in response to specific events. Emotions play a crucial role in numerous daily experiences of human beings and exert significant influence on their life, such as cognition, perception, and rational decision-making. Given the correlation between the two, emotion has been studied in psychology, philosophy, and neurobiology, which is consistent with the basis of emotion neuroscience (Panksepp, 2004). Although emotions are essential to human communication and interaction, until now, there has been no transparent, automated system for emotion recognition in human society. With the continuous development of artificial intelligence, the demand for emotional intelligence in the human-machine interface is increasingly amplified (Picard, 2003). Based on this, developing and implementing a new automatic emotional recognition algorithm becomes crucial. In this way, it is possible to improve and humanize the interaction between humans and machines. Many studies (Cloitre et al., 2019; Dickerson et al., 2020) have focused on detecting emotions by analyzing physical responses to emotional conditions. To this end, many studies have assessed the effects of emotion on different physiological variables, with particular efforts being made to evaluate EEG recordings (Alarcao and Fonseca, 2017). In recent decades, EEG-based emotion recognition research has gained popularity in many disciplines. However, available scientific data on emotional states and their structure are still limited. Researchers have verified that certain music can change neural activity and stimulate people's potential. Activity in the EEG correlates strongly with music-induced emotions. Vuilleumier and Trost (2015) shows that emotion recognition in music is a relatively rapid process.

Nordström and Laukka (2019) found that music-evoked emotions share some characteristics with basic emotions that map to valences observed in other emotional contexts (Vuilleumier and Trost, 2015). These findings suggest that emotional responses are multidimensional. Using functional magnetic resonance imaging (fMRI), Bodner and Shaw compared the changes in electrical signals in the brain as participants listened to Mozart's music and other music (Bodner et al., 2001). The results showed that in addition to the expected temporal lobe activation, the subjects listening to Mozart also activated the brain's frontal lobe, with significant α-wave changes. This phenomenon may be because Mozart's music is highly organized, and the regular repetition of melody is similar to the rhythmic cycle of brain electricity, which affects the human body. In addition, most previous studies on emotion recognition are based on body movement, posture, voice, and expression. However, tone and facial expressions can be deliberately hidden, so the corresponding credit is inaccurate. In contrast, EEG captures the electrical signals produced by neurons, and humans cannot control physiological signals on purpose, so EEG-based emotion recognition is more reliable.

Brain functional network studies include magnetic resonance imaging (MRI), functional magnetic resonance imaging (fMRI), and functional near-infrared spectroscopy (fNIRS). MRI is a very complex imaging examination. MRI provides in a particular brain, such as a tumor patient's. MRI shows only a still image of the brain, which is an anatomical image, with no actual brain activdes a map of the brain at a given moment. This structural information can be used to determine the size of a person's brain or whether there are abnormalitity. To get images of brain activity, fMRI is needed. FMRI can observe the specific brain activity clearly under test in the form of pictures, which has incomparable advantages. However, fMRI equipment will produce huge background noise when working, which seriously affects the effect of music appreciation. Meanwhile, fMRI has low time resolution, and the brain imaging obtained by fMRI is the average of brain activity within a few seconds. However, the brain responds effectively to stimuli within 30 ms, and the temporal resolution of fMRI makes it impossible to accurately analyze brain activity. fNIRS is the near-infrared spectroscopy (NIRS) used in functional neuroimaging. In application, the brain's response can be detected according to the behavior of neurons. It is a neuroimaging technique that uses spectroscopy to measure the level of brain activity. Functional near-infrared spectroscopy (FNIR) is a non-invasive functional brain imaging technique developed in recent years. But all of these methods are more expensive than EEG, require more training, and, most importantly, are less portable. And in the experiment, sometimes as long as you wear headphones, you can detect the quality of the data, so EEG is more widely used in the test.

The connection between music and the brain is interconnected. People with hearing impairments can't perceive pitch and rhythm correctly, but their brains are unaffected. Different music can trigger different emotions, and the same music can trigger other emotions, which has a lot to do with the current state of mind of the audience. In recent years, with the development of brain science, music, mood, and the correlation with EEG research have gradually become cutting-edge research in this area. With the help of EEG, research content can use machine learning algorithms to capture and study brain activity signals to predict emotional recognition patterns and translate them into commands. The development trend of EEG recognition of music arousing emotion is proposed. Based on this, this paper makes some conclusions about the brain mapping of music-induced emotion.

The main contributions of this paper are as follows:

- We give a detailed introduction to the music-based emotion recognition method and analyze the emotion analysis of EEG signals in detail.
- We introduced EEG-based music emotion recognition methods in detail, including data collection and processing, feature extraction, etc
- We discuss current challenges and future research directions for EEG-based music emotion.

The rest of the paper mainly includes some preliminary knowledge of music emotion recognition is introduced in Section 2. Then Section 3 reviews and summarizes the framework of EEG-based music emotion recognition. Section 4 discusses the current challenges and future work. Finally, we conclude this paper in Section 5.

## 2. Preliminary knowledge

With the rapid development of information science, various digital technologies are flourishing. Audio digitization has become an intermediate force in the digital trend. Audio is the result of digitizing and preserving sound, and music is an essential part of the audio. In recent years, digital music has attracted many scholars to research it. Compared with traditional music, digital music reflects its advantages in production, storage, dissemination, and retrieval, e.g., digital signal processing can reduce the cost of music storage and the application. At the same time, the popularization of the Internet has promoted the spread of digital music. With the development of audio retrieval technology, text content is unable to meet users' needs. In this context, digital audio content has become a hot spot of digital audio technology.

However, music's essential feature is based on emotion, and more music studies point out that emotion is a necessary criterion for people in music retrieval. The core of emotion-based music retrieval is music emotion recognition, which is the essential research direction of digital music. Music emotion recognition research covers musicology, psychology, music acoustics, audio signal processing, natural language processing, machine learning, and other fields. More than 100 related studies have shown that different listeners are generally consistent in judging the emotions expressed by music. Therefore, it is possible to perform emotion recognition with high accuracy.

Music emotion recognition began in the 1960s and has been around for decades. At the beginning of artificial intelligence, some people proposed the relationship between music content and emotion. Since then, universities and research institutes have conducted related research. In recent years, with the development of artificial intelligence, music emotion recognition research has progressed rapidly and has been successfully applied in various fields, e.g., music emotion retrieval, music art performance, intelligent space design, etc. Music emotion recognition technology is the research field of music visualization. It has laid the foundation with broad research prospects and essential application value.

In recent years, music emotion recognition has progressed rapidly and become one of the important research directions in digital music. In the current music emotion research, there are many problems need to be resolved, such as the scarcity of music emotion data sets, the difficulty of emotion quantification, and the limited accuracy of emotion recognition, with the test. This section will introduce the research status of music emotion recognition.

### 2.1. Music emotion recognition

Music emotion recognition is an interdisciplinary work that requires deep analysis and understanding of music, which not only involves signal processing and artificial intelligence but also needs to understand many fields such as auditory perception, psychology, and music theory. People's judgment of music emotion is subjective, and different experiences or ideas will influence the determination of music emotion. The characteristics of music, e.g., timbre, rhythm, and lyrics, also affect people's perception and judgment of musical emotion. Music expresses emotion with essential elements such as pitch, length, strength, and timbre, and how quantifying music's emotional characteristics is the key to solving the music emotion recognition problem.

Xia et al. (2012) used a continuous emotion mental model and a regression prediction model to generate robot dance movements based on constructing emotional changes and music beat sequences. Schmidt et al. (2010) chose a continuous emotion model to link the dynamic content of music with the acoustic feature model. Then the established regression model can study the emotional changes in music over time. Bresin and Friberg (2011) invited 20 music experts to express emotions such as happiness, sadness, fear, and calm by the numerical combination of 7 characteristic quantities in the device and obtained the relationship between usual quantities and musical emotions. Yang et al. (2006) used a continuous emotion mental model with regression modeling to predict the emotional value of music. Then, two fuzzy classifiers were used to measure the emotional intensity to identify the emotional content of the music. Sarkar and Saha (2015) using convolutional neural networks to identify music models and compared them with commonly classifiers, e.g., BP neural networks.

In recent years, music emotion recognition has received extensive attention. With the combination of deep learning methods, emotion recognition has been dramatically improved. However, music emotion recognition is a long-term task that still needs continuous innovation and improvement. As the in-depth application in various fields, music emotion recognition has created incomparable value, promoting the development of other areas. Music emotion recognition can accurately carry out personalized music recommendations. Adjusting the music according to the emotional needs of individuals can well solve the problem of individual differences and make music retrieval methods more diversified. With the further application of musical emotion recognition in the medical field, psychological therapy with music has become an effective treatment method in recent years. At the same time, music emotion recognition also plays an essential role in dealing with brain nerve problems. Therefore, the research on musical emotion recognition is of great significance to the in-depth development of various fields.

Music emotion recognition can accurately carry out personalized music recommendations. Adjusting the music according to the emotional needs of individuals can well solve the problem of individual differences and make music retrieval methods more diversified. With the further application of musical emotion recognition in the medical field, psychological therapy with music has become an effective treatment method in recent years. At the same time, music emotion recognition also plays an essential role in dealing with brain nerve problems. Therefore, the research on musical emotion recognition is of great significance to the in-depth development of various fields.

### 2.2. The processing of MER

**Figure 1:**
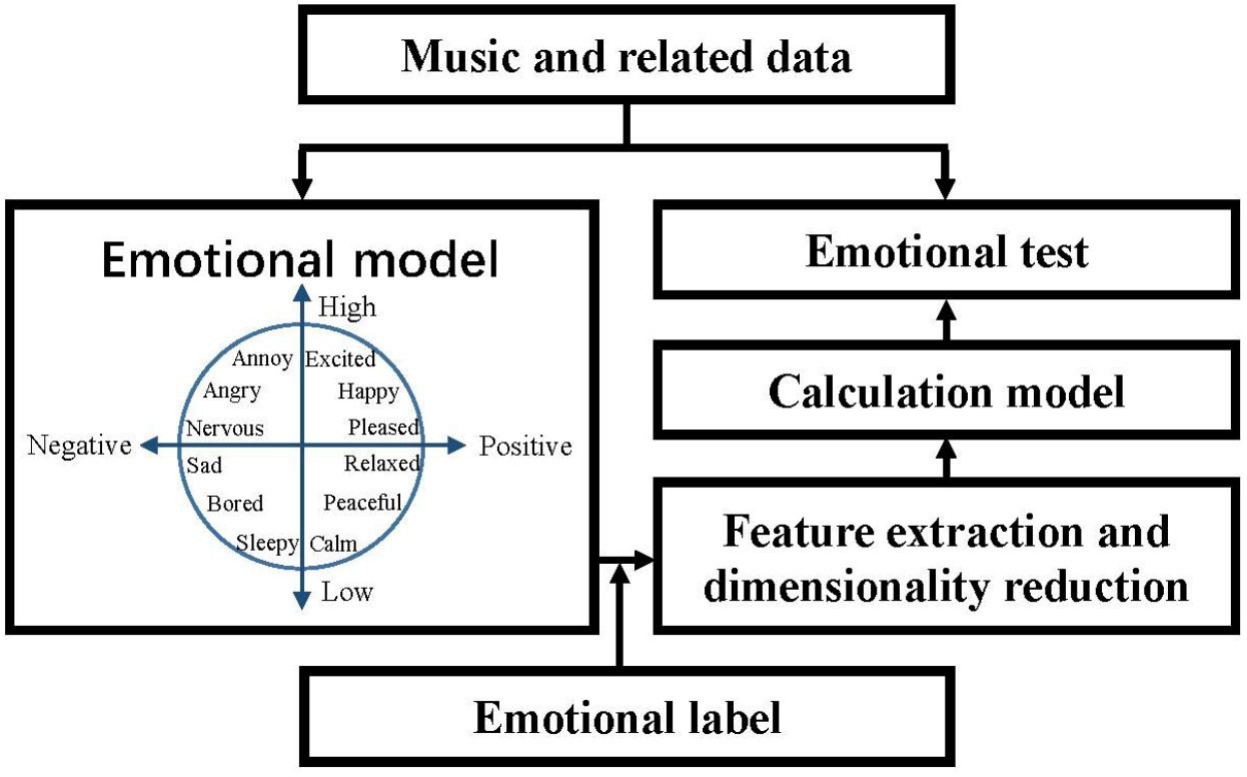
MER framework.

The existing MER algorithms are almost based on supervised learning. Therefore, establishing a learning library is necessary, i.e., music and related data, which is shown in Figure 1. Then, the music features are extracted by an emotional model, which forms feature vectors using dimensional reduction. This way, the music emotion recognition model is calculated by training the feature vectors and emotion labels. Finally, the performance feature is extracted from the unknown music, i.e., emotional test, and the classification result can be conducted by the trained recognition model.

### 2.3. MER models

The analysis and identification of music emotion require using a music emotion model. The music emotion model can effectively solve the problem that emotion is difficult to quantify. The basis for analyzing and identifying music emotion is selecting a suitable model according to the characteristics. The music sentiment analysis model can generally be divided into three parts, namely the music feature model, music emotion model, and cognitive classification model. Among them, the music emotion model is the basis for the final classification, and its selection is essential. Classical music emotion models include Hevner model (Hevner, 1936), Thayer model (Thayer, 1990), TWC model (Tellegen et al., 1999), and PAD model (Russell, 1980).

**Figure 2:**
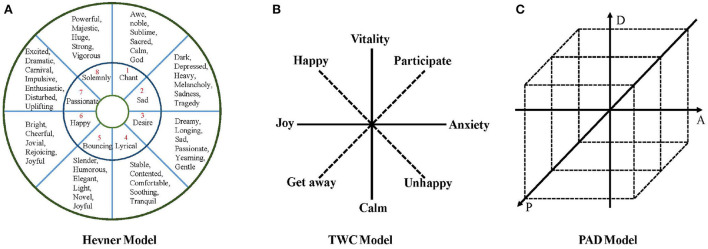
The common MER model. **(A)** Hevner model. **(B)** TWC model. **(C)** PAD model.

In computer music emotion analysis, the Hevner model is a commonly used psychological model of music emotion. As in Figure 2A, Hevner first proposed it in 1936. Hevner categorizes emotional adjectives into eight categories: solemn, sad, dreamy, quiet, graceful, happy, excited, and powerful. Each type of adjective is further subdivided into several more detailed and extensive emotional adjectives, totaling 67 words. The model combines musicology and psychology, is richer in selecting emotional keywords, and has a good effect on the emotional identification of musical works. Based on the Hevner model, Farnsworth (1954) found that several Hevner adjective clusters were not accurate enough to describe emotions and then effectively updated the model using 50 Hevner adjectives. The internal consistency was higher than in the original model, and the category distinction was better. In 2003, Schubert (2003) updated the Hevner model emotional adjective table, and the final list contained 46 words, which were divided into nine groups in the emotional space.

Thayer (1990) believes that the two underlying dimensions of emotion are two independent dimensions of arousal: energy awakening and tension awakening. Thayer's emotional model is two-dimensional, with pressure as the abscissa and energy as the ordinate. According to the power from calm to vitality and the force from happiness to anxiety, the plane is divided into four extreme areas, i.e., vitality, anxiety, contentment, and depression. The Thayer model is proposed from the perspective of psychology. It is described by dimensional thinking and can establish a good relationship with acoustic characteristics. Therefore, it is suitable for emotional recognition of audio music such as MP3 and WAV. However, it is still necessary to map to discrete emotion representations in the specific emotion recognition process. As shown in Figure 2B, Tellegen et al. (1999) revised and supplemented the Thayer model and proposed a TWC model, which used 38 adjectives to describe emotions, and added a set of coordinate systems based on the original two-dimensional coordinates. The horizontal and vertical coordinates are Happy and involved. The TWC model not only retains the Thayer model's natural and smooth emotional transition but also dramatically enriches the descriptions of musical emotions.

The PAD (Russell, 1980) three-dimensional effective model is a dimensional measurement model first proposed by Mehrabian and Russell and is widely used in psychology, marketing, and product satisfaction research, which is shown in Figure 2C. The model divides emotions into three dimensions: pleasure, activation, and dominance, where P represents pleasure, representing the positive and negative characteristics of an individual's emotional state, A means the degree of activation, representing the level of individual neurophysiological activation, and D represents dominance, representing the individual Control over the situation and others. PAD model regards the degree of emotion as a spatial angle coordinate system and divides feeling into three dimensions, pleasure, activation, and dominance. Among them, pleasure represents the trend of an individual's emotional state, activation degree represents the neurophysiological activation level of the individual, and dominance refers to the degree of domination of the individual relative to the situation and others. The model describes subtle emotional intensity through different coordinate points. Compared with traditional emotion description methods, the characteristics and advantages of the PAD model are that it can distinguish emotional states such as anger and fear. The PAD model can describe a state of emotional intensity according to specific spatial coordinates and define emotional words continuously and subtly. The Thayer model has only two dimensions and lacks the richness of emotions, while the Hevner emotional ring model classifies and describes emotions in too much detail. Except for the 8-dimensional emotional ring, each category is subdivided into several sub-categories. This classification is too fine-tuned, and complex semantic models are unnecessary for simple music with a single emotion.

In addition to the emotion model listed in Figure 2, some studies utilize probability distributions (Yang and Chen, 2011), rankings (Fan et al., 2017), and antonym pairs (Liu et al., 2019) to express musical emotion. Probability distributions represent the emotion of a song as a probability distribution in the emotion space, which can alleviate the problem of emotional subjectivity. Ranking and recommending music works according to emotional intensity can reduce the cognitive burden caused by continuous dynamic polarity and the inaccuracy caused by emotional subjectivity. Antonym pairs can make sentiment labels more objective. Figure 2 mainly introduces several classical music emotion models. The discrete model and multidimensional model are extensions of these methods.

#### 2.3.1. Discrete model

In the discrete emotion model research, experts, and scholars assume that all types of emotions can be described by a specific subset of primary emotional states. For example, Ekman's team believes there are only six basic emotions (Ekman et al., 1987), including anger, disgust, fear, happiness, sadness, and surprise. The number of basic emotions is fixed and limited, and there is no appropriate way to reflect the complexity and variability of emotions. In the research process, scholars found that the discrete emotion model is used to quantify the emotion type and intensity. However, it has to face many limitations when solving practical application problems. For example, Gelbrich (2010) found that negative emotions of anger were associated with intentions to complain and negative word of mouth, whereas frustration and helplessness were not.

#### 2.3.2. Multidimensional model

Another emotion model (Verma and Tiwary, 2017; Shu et al., 2018) provides a more effective way to quantify the types and intensity of emotions (Russell, 1980). The dimensional approach is based on the principle that human emotions can be represented as any point in two or three consecutive dimensions. One of the most prominent models is Russell's arousal valence emotion model. In this bipolar model, valence is represented by a positive or negative horizontal axis representing emotion. The vertical axis represents arousal, which describes the level of emotional activation. In this study, we used the arousal valence emotion model, which has been proven effective and reliable for identifying emotions during music listening, to convey human emotions (Salzman and Fusi, 2010).

### 2.4. Emotion feature

Music emotion classification is one of the tasks in the field of audio signal processing. The research work initially analyzed the emotional trend of audio from the perspective of audio signals, and solved the problem of music emotion classification by using common features such as timbre, dynamics and rhythm. Most of the methods used were some classic machine learning methods, which greatly improved the classification accuracy and efficiency. At present, there are two main methods for feature extraction. One is to train traditional machine learning (ML) models to predict emotions by extracting and using manual features. The other is doing it together through deep learning (DL) models.

- **Machine learning features** MER methods based on traditional machine learning can be divided into three categories, namely song-level classification MER, song-level dimension MER and dimension MEVD.
- **Song-level classification MER** Most song level MER classifications are based on mathematical-statistical models, of which K-nearest neighbors (KNN) and Support Vector Machine (SVM) are more common. In addition, decision tree (DT), random forest (RF), and Naive Bayes (NB) are also used for MER classification. MARSYAS (Li and Ogihara, 2003) extracted timbre, rhythm, and pitch features and input them into SVM for classification. However, the different emotion categories varied greatly. Yang et al. (2008a) evaluated various feature and fusion methods to improve classification accuracy through the late fusion of subtask merging. Liu et al. (2015) proposed Multi-emotional Similarity Preserving Embedding (ME-SPE), which combines ME-SPE with Calibration Label Ranking (CLR) to identify the emotion of music.
- **Song-level dimension MER** Commonly used MER regression algorithms include support vector regression (SVR), linear regression (LR), multiple linear regression (MLR), Gaussian process regression (GPR), and acoustic affective Gaussian (AEG). Yang et al. (2008b) take MER as a regression problem, extracting 114 audio features from existing toolkits and entering them into SVR. Malheiro et al. (2016) proposes three novel features of lyrics: slang presence, structural analysis, and semantic features. Wang et al. (2012) proposed a new generation model named Acoustic Emotional Gaussian (AEG) to identify emotional music, which shows higher accuracy than SVR and MLR models. Chen et al. (2017) discussed how to adjust AEG model to a personalized MER model with minimum user load. Chen et al. (2014a) explored the personalized MER using LR-based models. Fukayama and Goto (2016) input new acoustic signal into account using GPR. Soleymani et al. (2014) try static and dynamic methods by ML and DL methods.
- **Dimension MEVD** The idea of musical mood tracking was first proposed in Lu et al. (2005). Lu et al. (2005) divides the whole song into several independent segments and assigns an emotional label to each segment. The regression method can also be applied to MEVD but lacks time information. Schmidt et al. (2010) shows superior results in regression tasks of the spectral contrast features using multiple audio features. Xianyu et al. (2016) proposed DS-SVR method using two independent SVR models. One identifies mood changes between different songs, the other detects mood changes within a song, and then merges the results of the two SVRS into the final result.

### 2.5. Emotion evoking methods

In a broad sense, some stimuli can be classified as related to the organism's survival, while others are related to its growth. In this case, “emotional” stimuli are one such category, which creates a perception of understanding the situation and expresses the desired behavioral response accordingly. For example, galvanic skin response (GSR) evokes emotions by observing changes in electrical properties and autonomic nervous system activity (Das et al., 2016). In the current research on emotion recognition, various methods of emotion arousal (Widmann et al., 2018) have been developed. When we choose the proper arousal, it improves the accuracy of the data. Therefore, how to choose suitable and effective media to induce emotion experiments becomes the critical point of emotion recognition. By analyzing the source of emotional arousal materials, emotional arousal methods are usually divided into internal stimuli and external stimuli (Etkin et al., 2015; Alarcao and Fonseca, 2017). In the process of studying music to arouse emotions, experts and scholars also have their different views. Becker claimed that “music-induced emotional responses are not spontaneous” (Becker, 2001) and on the other hand, contrary to him, Peretz believed that “emotions are spontaneous and difficult to conceal” (Peretz, 2001). Noy's opinion is that “the emotions evoked by music are different from those generated in daily life and interpersonal communication” (Hunter and Schellenberg, 2010), and Peretz believes that “there is no theory that postulates this specificity of musical emotions” (Peretz, 2001).

### 2.6. Emotional information acquisition

In the experiments, it is not difficult to find that the results obtained by using multimedia emotional labels cannot be generalized to more interactive situations or everyday environments. To address this real-world problem, new research that uses interactive emotional stimuli to ensure the generality of Brain Computer Interfaces (BCI) results would be welcome (Pallavicini et al., 2018). Many experiments elicit emotions in different environments but don't use electroencephalography devices to record changes. Instead, other physiological indicators such as heart rate, electrical skin changes, and respiratory rate are collected. Conceptually, these paradigms could be helpful if replicated for EEG signal acquisition (Iacoviello et al., 2015).

Computer technology and emotion recognition algorithms are usually used when developing a human-computer emotional interaction system. Spontaneous physiological signals can be detected from a physiological perspective, allowing us to objectively and effectively analyze emotional states (Healey and Picard, 2005; Kreibig, 2010). When we want to know the activity of the cardiac autonomic nervous system corresponding to different emotional states, we can use the ECG to complete it (Agrafioti et al., 2011). Respiratory signals (RSP) can reveal a lot of information about emotions. For example, the frequency or depth of breathing is closely related to emotional changes (Zhang et al., 2017). However, during the experiment, the spontaneous physiological signals in the case will encounter problems such as no quantification standard and low classification accuracy when quantifying emotions (Fairclough, 2009; Nie et al., 2011). In processing automatic signals, EEG signals can provide a direct and comprehensive method for emotion recognition (Mauss and Robinson, 2009; Waugh et al., 2015).

### 2.7. EEG

EEG is a process of monitoring and recording information about the electrical activity of the human brain by generating spontaneous, rhythmic impulses from neurons in the brain. In neuroscience and psychology, EEG signals can describe the emotional state of the brain and human behavior, which can pick up subtle fluctuations in a person's emotional state. However, EEG signals are too weak to be recorded and are easily interfered by other physiological factors. In addition, to make the EEG signal non-linear, the original EEG signal needs to be preprocessed.

EEG has been widely used in clinical practice, and its characteristics can be summarized as follows:

- The EEG signal is feeble, with an amplitude around microvolts. Collecting EEG signals is easy to be interfered with by other signals. Therefore, the original EEG signal can be used for subsequent research after filtering, noise reduction, and further processing.
- The advantages of EEG signals are that they can directly reflect brain activity and change quickly. People's thinking, emotional changes, and other physiological factors can cause changes in EEG. An electroencephalogram differs from a typical electrocardiogram, a non-stationary and non-linear time series.

### 2.8. EEG datasets

With the rapid development of emotion recognition, a series of standardized emotion trigger databases have been established with corresponding emotion labels provided by psychologists. However, current research using music-based methods of emotional arousal is still limited without generally accepted standards. The researchers developed a number of mood databases on EEG. The benefit of open databases is that more standard methods can be used to verify the performance of emotional classification when different algorithms or classification models are used in experiments. Due to music copyright restrictions, some MER researchers use self-built and unpublished data sets. Table 1 lists some common data sets that are commonly used.

- **MediaEval**^1^ The data set contains randomly extracted 45-s musical snippets of complete songs. The 45 s excerpt (clip) is also annotated for the full length clip using a 9 point arousal and potency level. A set of features extracted by openSMILE are also available for data. The dataset initially had 1,000 creative general License songs annotated continuously (dynamically) on the wake and potency dimensions. We found some redundant songs and fixed some problems, which reduced the number of songs to 744.
- **CAL500**^2^ is a data set designed to evaluate music information retrieval systems. It consists of 502 songs from western pop music. Audio is represented as a time series of the first 13 Meir frequency cepstrum coefficients (and their first and second derivatives), extracted by sliding a 12 ms semi-overlapping short time window across the waveform of each song. Each song is annotated by at least 3 people and contains 135 music-related concepts covering six semantic categories.
- **CAL500exp**^3^ is a rich version of the CAL500 Music Information Retrieval dataset. CAL500exp is designed to facilitate automatic tagging of music on smaller time scales. The dataset consisted of the same songs, divided into 3,223 acoustically homogenous segments ranging in length from 3 to 16 s. Tags are annotated at the segment level rather than the track level. Notes are obtained from annotators with a strong musical background.
- **AMG 1608**^4^ It's a data set for emotional analysis of music. It contains frame-level acoustic features extracted from 1,608 30-s music clips and corresponding valency awakening (VA) notes provided by 665 subjects. The dataset consists of two parts: Campus subset. It is a subset of 240 songs annotated by 22 subjects from National Taiwan University and Academia Sinica. Amazon Mechanical Turk (AMT) subset. This subset contained 643 subjects using annotations for all 1,608 songs provided by the AMT each song received a total of 15 emotional annotations from each subject in this subset.
- **DEAM**^5^ The DEAM dataset consists of 1,802 fragments and complete songs annotated consecutively (per second) and for the entire song with valence and wake values. This dataset is a collection of data sets for the “Emotions in Music” task from the 2013–2015 MediaEval benchmark campaign, in addition to the original tags. The purpose of the emotional annotation collection is to detect the emotions expressed by music and musician composition content.
- **Soundtracks**^6^ The dataset provides complete audio files, documents, and behavioral ratings for the sharing of stimulating material for academic research. Audio files have been compressed into MP3 files using medium quality compression.
- **Emotify**^7^ The dataset contains extracts (1 min long) from 400 songs in 4 genres (rock, classical, pop, electronic). Notes were collected using the GEMS Scale (Geneva Emotional Music Scale). Each participant could choose up to three items from the scale (the strong emotions he felt while listening to the song).
- **DEAP**^8^ The most commonly used database for EEG emotion recognition is DEAP. The database was created by a consortium of four universities in the UK, the Netherlands and Sweden. The researchers collected physiological signals from 32 participants as they watched a 1-min music video and recorded the participants' SAM scale before and after the experiment (Koelstra et al., 2011). SAM scale was used to collect self-assessment of emotion in valence, arousal, dominance, and liking dimensions.

**Table 1:** A summary of datasets.

**Datasets**	**Conceptualization**	**Number of songs**	**Data type**	**Genres**
MediaEval	Dimensional	744	MP3	Rock, Pop, Soul, Blues
CAL500	Categorical	500	MP3	-
CAL500exp	Categorical	3,223	MP3	-
AMG 1608	Dimensional	1,608	MAV	Rock, Metal, Country, Jazz
DEAM	Dimensional	1,802	MP3	Rock, Pop, Electronic
Soundtracks	Both	360	MP3	Rap, R&B, Electronic
Emotify	Categorical	400	MP3	Rock, Classical, Pop, Electronic
DEAP	Dimensional	40	CSV	-

## 3. EEG-based music emotion recognition

With the emergence of advanced analytical tools, including mathematical models and brain-machine interface apparatus, the connectivity patterns between music and mood have attracted ever-lasting attention in the last decades. EEG plays a pivotal role in exploring the human body. EEG-based music emotion recognition combines the methods commonly used in processing EEG data and application in music emotion recognition. Other detection methods often describe the mental and physical changes of the human body by detecting the physiological response of the human body. An electrocardiogram (ECG) records changes in the heart's electrical activity during each cardiac cycle. Electromyogram (EMG) uses sensing electrodes to capture electrical signals from muscle impulses beneath the skin. Galvanic skin response (GSR) describes the mental state of the human body through the secretion of sweat glands. Electrooculography (EOG) records the electrical potential between the cornea and the retina of human eyes. Other detections such as speech, facial expression, heart rate, and respiratory rate are commonly used to profile emotional states. Of all the methods, EEG is the most used approach to physiological signals. EEG works by placing probe electrodes on a particular scalp and monitors the most complex response center of the human body indirectly, the brain. The most complex signals can be obtained, meaning that EEG signals contain valuable information. EEG-based music-induced emotion recognition is a method of great essence.

### 3.1. Data collection

Many factors have to be taken into consideration when acquiring MER data. This section presents an overview of common restrictions and unwritten rules followed by a mass of investigators. First of all, most subjects were between 20 and 30 years of age, and the ratio of men to women remained balanced. The subjects were also required to be free of brain diseases and mental illness. In music-related emotion studies, it is also necessary to exclude whether the subjects have received any professional training in music. Moreover, liking or familiarity with the music must also be considered in particular research tasks (Hadjidimitriou and Hadjileontiadis, 2012; Thammasan et al., 2017b; Naser and Saha, 2021). In addition, subjects were usually required to rest before data acquisition. Data acquisition is generally carried out in a dark and quiet environment, excluding interference from the external environment. A certain amount of participants, mainly from 9 to 27 in total, was usually allowed in a study. In another aspect, The choice of music was relatively personalized and varied considerably in different studies. An endeavor (Sangnark et al., 2021) have been made toward the perceptual distinctions from listening to two music types: music without lyrics (Melody) and music with lyrics (Song), which denoted that music of different forms could bring different emotional information within. Increasing numbers of researchers have been willing to using standard music databases for study as MER continues to develop, which eliminates the subjective operation of music selection and reduces the inconsistency of outcomes of different studies. Moreover, Some databases contain EEG data from subjects, which saves the experimenters from having to collect EEG data.

There are two main annotation methods for music emotion label annotation. The first one is Expert-based annotation. A given piece of music has been emotionally labeled, and the people's feelings are unique and subjective. The same song can bring different feelings to different people, which triggers the appearance of another criterion called Subject-based annotation. Subjects were asked to rate the degree of musical valence, arousal, and dominance. Target music selected from a set of music candidates shares a standard emotional label across all subjects, and the difference between different songs is significant enough simultaneously.

In different studies, the classification criteria of emotions vary according to the various tasks that researchers are interested in or the difficulty of the tasks. The most adopted is the two-dimensional emotion model. Self-evaluation reports, such as the Self-Assessment Manikin (SAM), are commonly used for evaluating a person's mental state in bipolar dimensions. The subjects were required to rate valence and arousal on a scale of 1 to 9. The higher the score, the stronger it is. There are usually four types: HVHA, HVLA, LVHA, and LVLA, namely happy, angry, relaxed, and sad. Some studies divide a single dimension into three categories: a score below three is considered low, and a score over six is considered high. In classification tasks, accuracy and F-score are generally used as model indicators. Unlike classification tasks, Sam Score can also be predicted by regression and other methods, while Root Mean Square Error (RMSE) is used as an indicator to evaluate the quality of the model.

### 3.2. Data preprocessing

EEG signal feature extraction has always been the key to music emotion recognition. Data preprocessing can transform complex EEG signals into data structures that are easier to understand and use. Different experiments used different pretreatment processes, which would affect the subsequent results. In general, according to the purpose of the pretreatment, the processing process can be divided into noise filtering and signal feature extraction.

Raw EEGs typically incorporate noise generated by eye movements, facial muscle movements, heart rate, and breathing. ICA (Independent Component Analysis) and Blind Source Separation commonly remove the above noise before processing EEG data. Another option is to filter out EEG bands of no interest. In the past few decades, EEGs have generally been classified into the following bands with different frequencies, i.e., δ (1–3 Hz), θ (4–7 Hz), α (8–13 Hz), β (14–30 Hz), and γ (31–50 Hz), as shown in Table 2. δ band is related to Deep sleep. β and γ bands are related to Relaxed. In many studies, δ and γ bands are usually excluded to reduce EMG and power line artifacts. EEG sequence is a typical time-varying signal, and a range of signal analysis techniques is proven to extract information from that effectively. The signal feature extraction method varies in different studies. Based on the above partial noise filtering methods, signal feature extraction can be divided into three parts: time, frequency, and time-frequency domain.

- **Time domain** Independent Component Analysis attempts to decompose a multivariate signal into independent non-Gaussian signals by maximizing the statistical independence of the estimated components. As mentioned above, it is applied to lessen the noise caused mainly by eye movement. Fractal dimensions, a widely used measure of complexity and irregularity, characterize a broad spectrum of objects, especially human physiology. The Higuchi algorithm is a selectable approach to calculating the FD value of EEG data and could give an outcome close to the theoretical value. Fluctuation Analysis is a universal analytic method of various fields for time-varying signals.
  Time series can generally decompose into trend, periodic, and random terms. Fluctuation analysis is a method used to judge whether the noise items in the time series have positive or negative self-correlation and whether the self-correlation is a long-range correlation. The detrended Fluctuation analysis is an improvement to the detrended analysis. The purpose is to eliminate the influence of the trend item on the detrended analysis.
  The entropy of the signal is a dimensionless indicator used to characterize the complexity of the signal sequence. The larger the entropy value, the greater the signal complexity. It is a big family containing approximate entropy, sample entropy, multiscale entropy, etc. Sample entropy is widely used in EEG signal processing because the calculation of sample entropy does not depend on data length and has a better consistency.
- **Frequency domain** The Frequency Domain refers to the analytic space in which mathematical functions or signals are conveyed in terms of frequency. Fourier transform and its variant, such as the short-time Fourier transform, are the most common paradigms to convert the time function into a set of sine waves representing different frequencies. Power spectral density has been adopted based on a fast Fourier transform with a window to obtain EEG signal frequency information.
- **Time-Frequency domain** Wavelet transform is a feature extraction algorithm combining the time and frequency domains. The wavelet decomposes the signal into different approximation and detail levels according to a specific frequency range while preserving the time information of the signal. The discrete wavelet transforms the signal into coarse approximations and details associated with low-pass and high-pass filters.
  Empirical Mode Decomposition decomposes the signal according to the time scale characteristics of the data itself. It does not need to set any basis function in advance, which is different from the Fourier decomposition methods based on a priori harmonic and wavelet basis functions. The EMD method can theoretically be applied to any signal decomposition, so it has obvious advantages in processing the non-stationary and non-linear signals with a high signal-to-noise ratio.

**Table 2:** A summary of EEG data.

**Frequency band**	**Frequency range (Hz)**	**Describe**
α	8–13	Appear in a relaxed state
β	14–30	Appear in a state of tension
δ	1–3	Appear in a state of extreme exertion
γ	31–50	Appear in a state of concentration
θ	4–7	Occurs in people with mental illness

### 3.3. Model collection

Sequentially, feature extraction is closely followed by model training. In this review, various relevant algorithms have been collected according to the taxonomy of machine learning. In most cases, MER is a label-available task. Commonly used classical classifiers or regressors, including General Linear Regression(GLR), Support Vector Machine(SVM), and Random Forest(RF), had yielded precise accuracy. SVM was the most used tool among all traditional algorithms and achieved remarkable prediction performance in different studies (Sourina et al., 2012; Thammasan et al., 2016c; Bo et al., 2019).

Radial basis function (RBF) kernel-based SVM can non-linearly map features onto a higher dimension space. It has been widely approved that kernel-based SVM has ensured better representation for samples and robustness. Thus, kernel-based SVM has been popular in MER (Thammasan et al., 2017a; Avramidis et al., 2021; Luo et al., 2022). Other machine learning methods like random forest (Pisipati and Nandy, 2021) and KNN (Bhatti et al., 2016) were also developed for MER. Fatemeh introduced a novel approach named Fuzzy Parallel Cascades outperformed the CNN-LSTM model.

Recently, with the trending usage of neural networks, deep learning-based algorithms, e.g., Multi-Layer Perception (MLP), Long Short-Term Memory (LSTM), have been referred to as better substitutes for traditional machine learning methods. In Rahman et al. (2020), MLP selected handcraft features as input. Convolution Neuron Network (CNN) is well-suited to processing image data with the bias of transitional invariance. Thus, the power spectrogram generated by the EEG frequency signal was adopted reasonably (Er et al., 2021; Liu et al., 2022). LSTM was born for time series data since it can keep track of arbitrary long-term dependencies in the input sequences. Luo et al. (2022) utilizes LSTM for sequence generation in his work. Additionally, deep learning algorithms overcome a troublesome and controversial problem, namely feature extraction, which liberates researchers from handcrafted feature selection to a certain extent. Panayu (Keelawat et al., 2019) used almost raw EEG signal as input into a 5-layer CNN without feature extraction and revealed supremacy compared to SVM. Sheykhivand et al. (2020) uses a fusion network of CNN and LSTM had been developed. Deep Belief Networks (Thammasan et al., 2016a) and Stacked Sparse Auto-Encoder (Li and Zheng, 2021) invented another path to solving MER.

### 3.4. EEG emotion recognition method

EEG is a physiological detection method mainly used to reflect brain activity. It has rich information on mental activity and is widely used by doctors and scientists to study brain function and diagnose neurological diseases. However, EEG signals generate a huge amount of data that can be difficult to analyze by observation during a study. Therefore, how to efficiently extract the required information from EEG signals has become the most urgent problem to be solved.

#### 3.4.1. EGG feature selection

High-dimensional EEG features may contain a large number of unnecessary features. In order to improve the classification performance, it is necessary to filter out the EEG features related to emotion before modeling the emotion classifier. Feature selection can be simply divided into supervised and unsupervised feature selection according to whether label information is used or not.

#### 3.4.2. Supervised feature selection

In supervised learning, Linear Discriminant Analysis (LDA) is a dimensionality reduction method for feature selection. The main idea of LDA is to find the appropriate projection direction according to the discriminative information of the class (Koelstra et al., 2011). The projection direction can be determined when the minimum within-class variance and the maximum within-class variance are simultaneously achieved. On the other hand, Maximum Relevance Minimum Redundancy (mRMR) is also an algorithm commonly used by supervised learning to obtain EEG features. It uses MI algorithm to select EEG features that can satisfy both maximum correlation and minimum redundancy (Atkinson and Campos, 2016).

#### 3.4.3. Unsupervised feature selection

Principal Component Analysis (PCA) is a commonly used method for unsupervised feature selection. By projecting the samples into a low-dimensional space, a series of linearly independent principal components are obtained. Principal component analysis preserves as much data information as possible by minimizing reconstruction errors during feature selection. K Nearest Neighbor (KNN) is also a non-parametric statistical method for classification and regression. Its core strategy is to identify k samples closest to the unknown sample point, and determine the classification information of the unknown samples from the majority of k samples (Hwang and Wen, 1998; Zhang et al., 2006). The selection of k value generally depends on the data used. Increasing k value in the classification task can suppress the interference of noise, but it will also blur the boundary between categories. Its advantages are easy to implement, small sample size and low computational complexity. It is relatively simple to implement and robust to noisy training data.

#### 3.4.4. EEG signal preprocessing and analysis

Since the architecture of the EEG headset is not exactly the same, and the cost of the equipment is also different, it is necessary to set different EEG equipment during the experiment. The main difference between these devices is the duration of time when the brainwave signals are collected, and the type of electrodes is also a factor (Chen et al., 2014b; Casson, 2019). Due to the sensitivity of the electrodes, the experiment required participants to remain still as they began collecting brainwave signals.

Over the past few years, there has been a lot of interest in using EEG signals to observe mood changes. In order to efficiently use EEG signals for emotion identification, the following steps are performed:

- Participants had to be tested for musical stimulation.
- The participants' brain voltage changes were observed and recorded throughout the experiment.
- Remove noise, shadow, and other interference items.
- The experimental results were analyzed and the eigenvalues were extracted.
- Training data, analyzing, and interpreting raw signals.

EEG signals have better temporal resolution than spatial resolution. In the process of music arousing emotion, the time change of electroencephalogram can be observed, including its amplitude and the change with time. In the process of EEG signal acquisition, due to the influence of environment and equipment, many noises are usually introduced, such as power frequency noise, ECG, EOG, and EMG caused by physiological signals of human body. In order to obtain relatively pure EEG data, it is necessary to preprocess the original EEG signal. Power frequency noise is mainly caused by the power supply of the device itself, and its frequency is 50 Hz. In the experimental process, the usual way is to use a 50Hz notch filter to work at 50 Hz frequency to remove the power frequency noise. In addition, the ECG is generated by the rhythmic operation of the heart, and the amplitude is large. However, the heart is far away from the electrode position, and the ECG signal is greatly weakened when it reaches the scalp. Therefore, this part is often ignored when we preprocess EEG signals.

The most widely used EEG analysis methods include four categories: time domain, frequency domain, time frequency domain, and non-linear method (Babloyantz et al., 1985; Phneah and Nisar, 2017).

(1) There are two main methods of time-domain EEG analysis: linear prediction and component analysis. Linear prediction is a linear combination of past output values with current and past input values. Component analysis is the mapping of datasets to feature sets for unsupervised learning.

(2) Spectrum analysis is to obtain frequency domain information in the EEG waveform through statistics and Fourier transform. Among them, power spectrum analysis is the most commonly used method.

### 3.5. Results and analysis

Works in MER were extremely hard to compare with each other owing to the processes of data acquisition and feature elicitation. In this part, we present the contribution of relevant research and shed light on the distinction between closely related jobs. Most studies focus on the improvement of prediction. Some others pay additional attention to feature selection for saving computational complexity or seek profound mechanisms inside. Tables 3, 4 present an overview of the scope and contributions of relevant works.

**Table 3:** Details of music emotion recognition algorithms based traditional method.

**Method**	**Dataset**	**Features**	**Classifier/regressor**	**Performance**
Avramidis et al. (2021)	DEAP	PSD, HFD, MFD, MADFA	RBF-SVM	Accuracy of 67% in Binary classification of arousal.
Hasanzadeh et al. (2021)	15 recruited listened to 7 songs	Spectrograms from Morlet wavelet transform	Fuzzy Parallel Cascades	2 types regression of Valence with RMSE 0.089.
Thammasan et al. (2016c)	15 recruited listened to 16 songs selected from MIDI	HFD	SVM	3% performance increase over the non-filtered.
Zainab and Majid (2021)	27 recruited listened to bilingual audio music of five genres	PSD, HFD, Hjorth Parameters. A series of linear measures of time domain	Hyper Pipes	Accuracy of 83.95% in quaternary classification..
Thammasan et al. (2016b)	12 recruited listened to 16 songs selected from MIDI	FD for EEG and handcraft feature for music	SVM	MCC of 84.17 and 90.25% in binary classification of arousal and valence, respectively.
Naser and Saha (2021)	DEAP	Wavelet transform, functional connectivity, graph-theory based features	RBF-SVM	Accuracy of arousal, valence, and dominance were 22.50, 14.87, and 19.44% above the empirical chance-level, respectively.
Thammasan et al. (2017b)	DEAP	PSD, HFD	Kernel SVM, MLP, Decision Tree	An average of 5% classification improvement of Unfamiliar set above familiar set in three methods.
Shahabi and Moghimi (2016)	19 recruited listened to six classical music excerpts	Connectivity matrices	SVM	Joyful vs. neutral, joyful vs. melancholic and familiar vs. unfamiliar trials reach accuracy of 93.7, 80.43, and 83.04%, respectively.
Lin et al. (2009)	26 recruited	PSD	One-against-one scheme SVM	Accuracy of 92.57% in quaternary classification.
Bhatti et al. (2016)	30 recruited listened to 4 genres of music	Latency to Amplitude Ratio, PSD, Wavelet transform	MLP, KNN, SVM	Accuracy of 78.11% (MLP) in quaternary classification.

**Table 4:** Details of music emotion recognition algorithms based deep learning method.

**Method**	**Dataset**	**Features**	**Classifier/regressor**	**Performance**
Keelawat et al. (2019)	12 recruited listened to 16 songs selected from MIDI	Segmented EEG	CNN	Accuracy of 78.36 and 83.67% in binary classification of arousal and valence, respectively.
Er et al. (2021)	Nine recruited listened to 16 audio tracks	Power spectrogram	Pretrained VGG16	Accuracy of 73.28% in quaternary classification.
Thammasan et al. (2016a)	15 recruited listened to 16 songs selected from MIDI	HFD, PSD, Discrete Wavelet Transform	Deep Belief Networks	Accuracy of 81.98% in binary classification of arousal and valence.
Rahman et al. (2020)	24 recruited listened to Twelve songs	DFA, Approximate Entropy, Fuzzy Entropy, Shannon's Entropy, Permutation Entropy, Hjorth Parameters, Hurst Exponent	Neuron Network	3 emotion scales (Depressing vs. Exciting and Sad vs. Happy and Irritating vs. Soothing).
Liu et al. (2022)	15 recruited listened to 13 music excerpts	Power spectrogram	Xception	Accuracy of 76.84% in HVHA vs. LVLA
Luo et al. (2022)	DEAP	PSD	RBF-SVM, LSTM	A SAM score of 6.17(high) and 4.76(low) in continuous valance scale, that is close to 6.98 and 4.36 evaluated in music database.
Hsu et al. (2018)	IADS	Segmented EEG	Neuron Network	MSE of 1.865 in 2D continuous SAM score.
Sheykhivand et al. (2020)	16 recruited listened to ten music excerpts	Segmented EEG	CNN, LSTM	Accuracy of 76.84% in HVHA vs. LVLA.
Li and Zheng (2021)	21 recruited listened to 15 music excerpts	Segmented EEG	Stacked Sparse Auto-Encoder	Accuracy of 59.5% and 66.8% in binary classification of arousal and valence, respectively.
Salama et al. (2018)	DEAP	Segmented EEG	3D CNN	Accuracy of 88.49% and 87.44% in binary classification of arousal and valence, respectively.

To summarize the works mentioned above, this article divides them into four categories regarding their focus and contributions.

#### 3.5.1. Performance comparison

Many works have listed a series of algorithms. Therefore, differences can be quickly concluded by their achievements in prediction results. Diverse versions of SVMs have been investigated in Lin et al. (2009). In Bhatti et al. (2016), MLP had been proven to outperform KNN and SVM in MER. A comparison to random forest and multilayer perceptron shows slight superiority of hyper pipes (Zainab and Majid, 2021). In addition, model selection is time-dependent. In the early years of MER, shallow machine learning methods, e.g., SVM, kernel SVM, KNN, and RF, were widely adopted. As time went by, algorithms went more profound and more complex, like CNN (Salama et al., 2018; Keelawat et al., 2019; Er et al., 2021), LSTM (Sheykhivand et al., 2020), and SSAE (Li and Zheng, 2021), and promising performances have been witnessed.

#### 3.5.2. Features selection preference

Generally, linear features like Mean, Standard Deviation are considered insufficient for precise prediction. Therefore, non-linear hand-crafted features showed up in the traditional machine learning methods. Several taxonomies of feature elicitation patterns have been broadly endorsed. Most works set a high value on FD in the time domain. The entropy of many forms of EEG signal was also available in predicting emotion. Undoubtedly, PSD is the most used method in the frequency domain and Wavelet Transform is pivotal in the time-frequency domain. However, earthshaking change have taken place as deep learning based algorithms began to flourish in MER, which diminished the importance of feature selection largely.

#### 3.5.3. Accessory effective factors

Many works also took liking or familiarity into consideration because whether a person enjoys or gets familiar with a specific song would have an impact on the correctness of prediction to a great extent. Such investigation has been carried out by Naser (Naser and Saha, 2021), who found that testing in low-liking music could perform better than high-like. In Thammasan et al. (2017b), the same phenomenon can be observed. Familiarity effects in MER have been proven to impair the capacity of the model to predict. Both observations indicate that liking or familiarity would impact emotion recognition.

#### 3.5.4. Multi-modal fusion

Despite a series of efforts have been exerted in algorithm melioration, another promising path had been opened up for better performance, that is multi-modal fusion. Multi-modal is a trend in future works owing to the shortness of EEG signals, which is too complex to understand for a machine, speaking of only a few data available for training in a very single experiment. Data from other pathways are welcome for affective recognition that would improve performance. Like Nattapong (Thammasan et al., 2016b) did in his work, he fused features from both EEG and music and reached higher performance.

#### 3.5.5. Applications

Music emotional recognition is an interdisciplinary field that spans medical psychology and computer science. Human's affection could be precisely detected by machine. By this way, it's certified that the inner alteration in mood could be effected by the external music. The quantified emotional score could be viewed as a criterion in music therapy. A bulk of jobs (Shahabi and Moghimi, 2016; Thammasan et al., 2017b) centered on the brain communication by analyzing connectivity among disparate EEG channels when exposed to music stimulation, which attempted to uncover the function of areas of brain. Interestingly, A reverse research have been conducted that generated emotion-related music by feeding neuron network with EEG signal (Li and Zheng, 2021).

## 4. Discussion and future work

Music emotion recognition is an interdisciplinary subject with a wide range of applications. Many researchers have carried on the in-depth discussion on it and have made some achievements. However, music emotion recognition is still in the process of development and should be further explored. The subjectivity of music emotion has a significant influence on the expression of emotion. In analyzing music emotion, studying its characteristics is a basic premise for emotional calculation. It is not trivial for researchers to obtain objective and accurate emotional expression and establish effective and high-quality teaching resources because of the subjective nature of the expression and emotion inspired by music. Recently, music emotion recognition-based EEG is an emerging topic in affective computing and social signal processing, gaining more attention. Development in this field is to meet the demand of People's Daily lives, such as in human-computer interaction, the machine can communicate and understand humans.

In the future, multimodal-signals-based MER can be studied. For example, music emotion analysis based on physiological and EEG signals can obtain more comprehensive and practical information by analyzing the correlation between EEG signals and physiological signals. At the same time, signal conversion based on music sentiment analysis is also a promising research direction. The mutual conversion between EEG signals and physiological signals is realized through music emotion analysis.

## 5. Conclusion

Music plays an essential role in People's Daily life. It is necessary to relax their emotions and regulate their physical and mental health. It can affect people's emotions, intelligence, and psychology. As the research progressed, the researchers began to explore what happens to the body during listening to music and the association between different music and induced emotions. EEG is one of the physiological signals of the human body, which contains rich physiological and disease information and is widely used in clinical practice.

## Author contributions

YW, JW, and ZY performed the literature search. XC provided facilities and support. All authors contributed to the article and approved the submitted version.

## Funding

This work was supported by the Ningbo Public Welfare Technology Plan Project (2019C50081), the Shenzhen Governmental Science and Technology for Basic Research Program Grant (JCYJ20180507182446643), the Shenzhen Governmental Science and Technology for Shenzhen-Hong Kong Joint Program Grant (SGDX20201103095406023), the Guangdong Province Science and Technology Program Grant (2018A050501010), the Natural Science Foundation of Fujian Province of China (No. 2019J01573), the Shenzhen Science and Technology for International Cooperation Research Grant (GJHZ20190821155201661), and SIAT Innovation Program for Excellent Young Researchers (E1G062, E2G037).

## Conflict of interest

The authors declare that the research was conducted in the absence of any commercial or financial relationships that could be construed as a potential conflict of interest.

## Publisher's note

All claims expressed in this article are solely those of the authors and do not necessarily represent those of their affiliated organizations, or those of the publisher, the editors and the reviewers. Any product that may be evaluated in this article, or claim that may be made by its manufacturer, is not guaranteed or endorsed by the publisher.

## Footnotes

^1^ http://www.multimediaeval.org/mediaeval2015/emotioninmusic2015/

^2^ https://paperswithcode.com/dataset/cal500

^3^ https://paperswithcode.com/dataset/cal500exp

^4^ http://amg1608.blogspot.com/

^5^ https://cvml.unige.ch/databases/DEAM/

^6^ https://osf.io/p6vkg/

^7^ http://www2.projects.science.uu.nl/memotion/emotifydata/

^8^ http://www.eecs.qmul.ac.uk/mmv/datasets/deap/

## References

[B1] Agrafioti F. Hatzinakos D. Anderson A. K. (2011). ECG pattern analysis for emotion detection. IEEE Trans. Affect. Comput. 3, 102–115. 10.1109/T-AFFC.2011.28

[B2] Alarcao S. M. Fonseca M. J. (2017). Emotions recognition using EEG signals: a survey. IEEE Trans. Affect. Comput. 10, 374–393. 10.1109/TAFFC.2017.2714671

[B3] Atkinson J. Campos D. (2016). Improving BCI-based emotion recognition by combining EEG feature selection and kernel classifiers. Expert Syst. Appl. 47, 35–41. 10.1016/j.eswa.2015.10.049

[B4] Avramidis K. Zlatintsi A. Garoufis C. Maragos P. (2021). “Multiscale fractal analysis on EEG signals for music-induced emotion recognition,” in 2021 29th European Signal Processing Conference (EUSIPCO) (Dublin: IEEE), 1316–1320. 10.23919/EUSIPCO54536.2021.9616140

[B5] Babloyantz A. Salazar J. Nicolis C. (1985). Evidence of chaotic dynamics of brain activity during the sleep cycle. Phys. Lett. A 111, 152–156. 10.1016/0375-9601(85)90444-X

[B6] Becker J. (2001). “Anthropological perspectives on music and emotion,” in Music and Emotion: Theory and Research, eds P. N. Juslin and J. A. Sloboda (New York, NY: Oxford University Press), 135–160.

[B7] Bhatti A. M. Majid M. Anwar S. M. Khan B. (2016). Human emotion recognition and analysis in response to audio music using brain signals. Comput. Hum. Behav. 65, 267–275. 10.1016/j.chb.2016.08.029

[B8] Bo H. Ma L. Liu Q. Xu R. Li H. (2019). Music-evoked emotion recognition based on cognitive principles inspired EEG temporal and spectral features. Int. J. Mach. Learn. Cybernet. 10, 2439–2448. 10.1007/s13042-018-0880-z

[B9] Bodner M. Muftuler L. T. Nalcioglu O. Shaw G. L. (2001). FMRI study relevant to the mozart effect: brain areas involved in spatial-temporal reasoning. Neurol. Res. 23, 683–690. 10.1179/016164101101199108 11680506

[B10] Bresin R. Friberg A. (2011). Emotion rendering in music: range and characteristic values of seven musical variables. Cortex 47, 1068–1081. 10.1016/j.cortex.2011.05.009 21696717

[B11] Cacioppo J. T. Freberg L. Cacioppo S. (2021). Discovering Psychology: The Science of Mind. Boston, MA: Cengage Learning.

[B12] Casson A. J. (2019). Wearable EEG and beyond. Biomed. Eng. Lett. 9, 53–71. 10.1007/s13534-018-00093-6 30956880 PMC6431319

[B13] Chen Y.-A. Wang J.-C. Yang Y.-H. Chen H. (2014a). “Linear regression-based adaptation of music emotion recognition models for personalization,” in 2014 IEEE International Conference on Acoustics, Speech and Signal Processing (ICASSP) (Florence: IEEE), 2149–2153. 10.1109/ICASSP.2014.6853979

[B14] Chen Y.-A. Wang J.-C. Yang Y.-H. Chen H. H. (2017). Component tying for mixture model adaptation in personalization of music emotion recognition. IEEE/ACM Trans. Audio Speech Lang. Process. 25, 1409–1420. 10.1109/TASLP.2017.2693565

[B15] Chen Y.-H. De Beeck M. O. Vanderheyden L. Carrette E. Mihajlović V. Vanstreels K.. (2014b). Soft, comfortable polymer dry electrodes for high quality ECG and EEG recording. Sensors 14, 23758–23780. 10.3390/s141223758 25513825 PMC4299086

[B16] Cloitre M. Khan C. Mackintosh M.-A. Garvert D. W. Henn-Haase C. M. Falvey E. C.. (2019). Emotion regulation mediates the relationship between aces and physical and mental health. Psychol. Trauma Theory Res. Pract. Policy 11, 82. 10.1037/tra0000374 29745688

[B17] Das P. Khasnobish A. Tibarewala D. (2016). “Emotion recognition employing ECG and GSR signals as markers of ANS,” in 2016 Conference on Advances in Signal Processing (CASP) (Pune: IEEE), 37–42. 10.1109/CASP.2016.7746134

[B18] Dickerson K. L. Skeem J. L. Montoya L. Quas J. A. (2020). Using positive emotion training with maltreated youths to reduce anger bias and physical aggression. Clin. Psychol. Sci. 8, 773–787. 10.1177/2167702620902118 34136312 PMC8204906

[B19] Ekman P. Friesen W. V. O'sullivan M. Chan A. Diacoyanni-Tarlatzis I. Heider K.. (1987). Universals and cultural differences in the judgments of facial expressions of emotion. J. Pers. Soc. Psychol. 53, 712. 10.1037/0022-3514.53.4.712 3681648

[B20] Er M. B. Çiğ H. Aydilek İ. B. (2021). A new approach to recognition of human emotions using brain signals and music stimuli. Appl. Acoust. 175, 107840. 10.1016/j.apacoust.2020.10784035585922

[B21] Etkin A. Büchel C. Gross J. J. (2015). The neural bases of emotion regulation. Nat. Rev. Neurosci. 16, 693–700. 10.1038/nrn4044 26481098

[B22] Fairclough S. H. (2009). Fundamentals of physiological computing. Interact. Comput. 21, 133–145. 10.1016/j.intcom.2008.10.011

[B23] Fan J. Tatar K. Thorogood M. Pasquier P. (2017). “Ranking-based emotion recognition for experimental music,” in ISMIR (Suzhou: International Society for Music Information Retrieval), 368–375.

[B24] Farnsworth P. R. (1954). A study of the Hevner adjective list. J. Aesthet. Art Crit. 13, 97–103. 10.1111/1540_6245.jaac13.1.0097

[B25] Fukayama S. Goto M. (2016). “Music emotion recognition with adaptive aggregation of Gaussian process regressors,” in 2016 IEEE International Conference on Acoustics, Speech and Signal Processing (ICASSP) (Shanghai: IEEE), 71–75. 10.1109/ICASSP.2016.7471639

[B26] Gelbrich K. (2010). Anger, frustration, and helplessness after service failure: coping strategies and effective informational support. J. Acad. Market. Sci. 38, 567–585. 10.1007/s11747-009-0169-6

[B27] Hadjidimitriou S. K. Hadjileontiadis L. J. (2012). Toward an EEG-based recognition of music liking using time-frequency analysis. IEEE Trans. Biomed. Eng. 59, 3498–3510. 10.1109/TBME.2012.2217495 23033323

[B28] Hasanzadeh F. Annabestani M. Moghimi S. (2021). Continuous emotion recognition during music listening using EEG signals: a fuzzy parallel cascades model. Appl. Soft Comput. 101, 107028. 10.1016/j.asoc.2020.107028

[B29] Healey J. A. Picard R. W. (2005). Detecting stress during real-world driving tasks using physiological sensors. IEEE Trans. Intell. Transport Syst. 6, 156–166. 10.1109/TITS.2005.848368

[B30] Hevner K. (1936). Experimental studies of the elements of expression in music. Am. J. Psychol. 48, 246–268. 10.2307/1415746

[B31] Hsu J.-L. Zhen Y.-L. Lin T.-C. Chiu Y.-S. (2018). Affective content analysis of music emotion through EEG. Multimedia Syst. 24, 195–210. 10.1007/s00530-017-0542-029367844

[B32] Hunter P. G. Schellenberg E. G. (2010). “Music and emotion,” in Music Perception (New York, NY: Springer), 129–164. 10.1007/978-1-4419-6114-3_5

[B33] Hwang W. J. Wen K. W. (1998). Fast KNN classification algorithm based on partial distance search. Electron. Lett. 34. 2062–2063. 10.1049/el:19981427

[B34] Iacoviello D. Petracca A. Spezialetti M. Placidi G. (2015). A real-time classification algorithm for EEG-based BCI driven by self-induced emotions. Comput. Methods Prog. Biomed. 122, 293–303. 10.1016/j.cmpb.2015.08.011 26358282

[B35] Keelawat P. Thammasan N. Numao M. Kijsirikul B. (2019). Spatiotemporal emotion recognition using deep CNN based on EEG during music listening. arXiv preprint arXiv:1910.09719.

[B36] Koelstra S. Muhl C. Soleymani M. Lee J.-S. Yazdani A. Ebrahimi T.. (2011). DEAP: a database for emotion analysis; using physiological signals. IEEE Trans. Affect. Comput. 3, 18–31. 10.1109/T-AFFC.2011.15

[B37] Kreibig S. D. (2010). Autonomic nervous system activity in emotion: a review. Biol. Psychol. 84, 394–421. 10.1016/j.biopsycho.2010.03.010 20371374

[B38] Li T. Ogihara M. (2003). “Detecting emotion in music,” in 4th International Conference on Music Information Retrieval (Baltimore, MD).

[B39] Li Y. Zheng W. (2021). Emotion recognition and regulation based on stacked sparse auto-encoder network and personalized reconfigurable music. Mathematics 9, 593. 10.3390/math9060593

[B40] Lin W.-C. Chiu H.-W. Hsu C.-Y. (2006). “Discovering EEG signals response to musical signal stimuli by time-frequency analysis and independent component analysis,” in 2005 IEEE Engineering in Medicine and Biology 27th Annual Conference (Shanghai: IEEE), 2765–2768. 10.1109/IEMBS.2005.1617045 17282814

[B41] Lin Y.-P. Wang C.-H. Jung T.-P. Wu T.-L. Jeng S.-K. Duann J.-R.. (2010). EEG-based emotion recognition in music listening. IEEE Trans. Biomed. Eng. 57, 1798–1806. 10.1109/TBME.2010.2048568 20442037

[B42] Lin Y.-P. Wang C.-H. Wu T.-L. Jeng S.-K. Chen J.-H. (2009). “EEG-based emotion recognition in music listening: a comparison of schemes for multiclass support vector machine,” in 2009 IEEE International Conference on Acoustics, Speech and Signal Processing (Taipei: IEEE), 489–492. 10.1109/ICASSP.2009.4959627

[B43] Liu H. Fang Y. Huang Q. (2019). “Music emotion recognition using a variant of recurrent neural network,” in 2018 International Conference on Mathematics, Modeling, Simulation and Statistics Application (MMSSA 2018) (Shanghai: Atlantis Press), 15–18. 10.2991/mmssa-18.2019.4

[B44] Liu J. Sun L. Huang M. Xu Y. Li R. (2022). Enhancing emotion recognition using region-specific electroencephalogram data and dynamic functional connectivity. Front. Neurosci. 16, 884475. 10.3389/fnins.2022.884475 35585922 PMC9108496

[B45] Liu Y. Liu Y. Zhao Y. Hua K. A. (2015). What strikes the strings of your heart?–Feature mining for music emotion analysis. IEEE Trans. Affect. Comput. 6, 247–260. 10.1109/TAFFC.2015.2396151

[B46] Lu L. Liu D. Zhang H.-J. (2005). Automatic mood detection and tracking of music audio signals. IEEE Trans. Audio Speech Lang. Process. 14, 5–18. 10.1109/TSA.2005.860344

[B47] Luo G. Chen H. Li Z. Wang M. (2022). “Music generation based on emotional EEG,” in 2022 the 6th International Conference on Innovation in Artificial Intelligence (ICIAI) (Harbin), 143–147. 10.1145/3529466.3529492

[B48] Malheiro R. Panda R. Gomes P. Paiva R. P. (2016). Emotionally-relevant features for classification and regression of music lyrics. IEEE Trans. Affect. Comput. 9, 240–254. 10.1109/TAFFC.2016.2598569

[B49] Mauss I. B. Robinson M. D. (2009). Measures of emotion: a review. Cogn. Emot. 23, 209–237. 10.1080/02699930802204677 19809584 PMC2756702

[B50] Naser D. S. Saha G. (2021). Influence of music liking on EEG based emotion recognition. Biomed. Signal Process. Control 64, 102251. 10.1016/j.bspc.2020.102251

[B51] Nie D. Wang X.-W. Shi L.-C. Lu B.-L. (2011). “EEG-based emotion recognition during watching movies,” in 2011 5th International IEEE/EMBS Conference on Neural Engineering (Boston, MA: IEEE), 667–670. 10.1109/NER.2011.5910636

[B52] Nordström H. Laukka P. (2019). The time course of emotion recognition in speech and music. J. Acoust. Soc. Am. 145, 3058–3074. 10.1121/1.5108601 31153307

[B53] Pallavicini F. Ferrari A. Pepe A. Garcea G. Zanacchi A. Mantovani F. (2018). “Effectiveness of virtual reality survival horror games for the emotional elicitation: preliminary insights using resident evil 7: biohazard,” in International Conference on Universal Access in Human-Computer Interaction (Las Vegas, NV: Springer), 87–101. 10.1007/978-3-319-92052-8_8

[B54] Panksepp J. (2004). Affective Neuroscience: The Foundations of Human and Animal Emotions. New York, NY: Oxford University Press. 10.1176/appi.ajp.159.10.1805

[B55] Peretz I. (2001). “Listen to the brain. A biological perspective on musical emotions,” in Music and Emotion: Theory and Research, eds P. N. Juslin and J. A. Sloboda (New York, NY: Oxford University Press), 105–134.

[B56] Phneah S. W. Nisar H. (2017). EEG-based alpha neurofeedback training for mood enhancement. Austral. Phys. Eng. Sci. Med. 40, 325–336. 10.1007/s13246-017-0538-2 28290068

[B57] Picard R. W. (2003). Affective computing: challenges. Int. J. Hum. Comput. Stud. 59, 55–64. 10.1016/S1071-5819(03)00052-1

[B58] Pisipati M. Nandy A. (2021). “Human emotion recognition using EEG signal in music listening,” in 2021 IEEE 18th India Council International Conference (INDICON) (Guwahati: IEEE), 1–6. 10.1109/INDICON52576.2021.9691724

[B59] Rahman J. S. Gedeon T. Caldwell S. Jones R. (2020). “Brain melody informatics: analysing effects of music on brainwave patterns,” in 2020 International Joint Conference on Neural Networks (IJCNN) (IEEE), 1–8. 10.1109/IJCNN48605.2020.9207392

[B60] Russell J. A. (1980). A circumplex model of affect. J. Pers. Soc. Psychol. 39, 1161. 10.1037/h0077714

[B61] Salama E. S. El-Khoribi R. A. Shoman M. E. Shalaby M. A. W. (2018). EEG-based emotion recognition using 3D convolutional neural networks. Int. J. Adv. Comput. Sci. Appl. 9, 329–337. 10.14569/IJACSA.2018.090843

[B62] Salzman C. D. Fusi S. (2010). Emotion, cognition, and mental state representation in amygdala and prefrontal cortex. Annu. Rev. Neurosci. 33, 173. 10.1146/annurev.neuro.051508.135256 20331363 PMC3108339

[B63] Sangnark S. Autthasan P. Ponglertnapakorn P. Chalekarn P. Sudhawiyangkul T. Trakulruangroj M.. (2021). Revealing preference in popular music through familiarity and brain response. IEEE Sens. J. 21, 14931–14940. 10.1109/JSEN.2021.3073040

[B64] Sarkar R. Saha S. K. (2015). “Music genre classification using emd and pitch based feature,” in 2015 Eighth International Conference on Advances in Pattern Recognition (ICAPR) (Kolkata: IEEE), 1–6. 10.1109/ICAPR.2015.7050714

[B65] Schmidt E. M. Turnbull D. Kim Y. E. (2010). “Feature selection for content-based, time-varying musical emotion regression,” in Proceedings of the International Conference on Multimedia Information Retrieval, 267–274. 10.1145/1743384.1743431

[B66] Schubert E. (2003). Update of the hevner adjective checklist. Percept. Motor Skills, 96(3_Suppl), 1117–1122. 10.2466/pms.2003.96.3c.1117 12929763

[B67] Shahabi H. Moghimi S. (2016). Toward automatic detection of brain responses to emotional music through analysis of EEG effective connectivity. Comput. Hum. Behav. 58, 231–239. 10.1016/j.chb.2016.01.005

[B68] Sheykhivand S. Mousavi Z. Rezaii T. Y. Farzamnia A. (2020). Recognizing emotions evoked by music using cnn-lstm networks on EEG signals. IEEE Access 8, 139332–139345. 10.1109/ACCESS.2020.3011882

[B69] Shu L. Xie J. Yang M. Li Z. Li Z. Liao D.. (2018). A review of emotion recognition using physiological signals. Sensors 18, 2074. 10.3390/s18072074 29958457 PMC6069143

[B70] Soleymani M. Aljanaki A. Yang Y.-H. Caro M. N. Eyben F. Markov K.. (2014). “Emotional analysis of music: a comparison of methods,” in Proceedings of the 22nd ACM International Conference on Multimedia (Orlando, FL), 1161–1164. 10.1145/2647868.2655019

[B71] Sourina O. Liu Y. Nguyen M. K. (2012). Real-time EEG-based emotion recognition for music therapy. J. Multimodal User Interfaces 5, 27–35. 10.1007/s12193-011-0080-6

[B72] Tellegen A. Watson D. Clark L. A. (1999). On the dimensional and hierarchical structure of affect. Psychol. Sci. 10, 297–303. 10.1111/1467-9280.00157

[B73] Thammasan N. Fukui K.-I. Numao M. (2016a). “Application of deep belief networks in EEG-based dynamic music-emotion recognition,” in 2016 International Joint Conference on Neural Networks (IJCNN) (Vancouver, BC: IEEE), 881–888. 10.1109/IJCNN.2016.7727292

[B74] Thammasan N. Fukui K.-I. Numao M. (2016b). Fusion of EEG and musical features in continuous music-emotion recognition. arXiv preprint arXiv:1611.10120.

[B75] Thammasan N. Fukui K.-I. Numao M. (2016c). “An investigation of annotation smoothing for EEG-based continuous music-emotion recognition,” in 2016 IEEE International Conference on Systems, Man, and Cybernetics (SMC), 3323–3328. Budapest: IEEE. 10.1109/SMC.2016.7844747

[B76] Thammasan N. Fukui K.-I. Numao M. (2017a). “Multimodal fusion of EEG and musical features in music-emotion recognition,” in Proceedings of the AAAI Conference on Artificial Intelligence (San Francisco, CA). 10.1609/aaai.v31i1.11112

[B77] Thammasan N. Moriyama K. Fukui K.-I. Numao M. (2017b). Familiarity effects in EEG-based emotion recognition. Brain informatics 4, 39–50. 10.1007/s40708-016-0051-5 27747819 PMC5319949

[B78] Thayer R. E. (1990). The Biopsychology of Mood and Arousal. Chicago: Oxford University Press. 10.1086/417761

[B79] Verma G. K. Tiwary U. S. (2017). Affect representation and recognition in 3d continuous valence-arousal-dominance space. Multimedia Tools Appl. 76, 2159–2183. 10.1007/s11042-015-3119-y

[B80] Vuilleumier P. Trost W. (2015). Music and emotions: from enchantment to entrainment. Ann. N. Y. Acad. Sci. 1337, 212–222. 10.1111/nyas.12676 25773637

[B81] Wang J.-C. Yang Y.-H. Wang H.-M. Jeng S.-K. (2012). “The acoustic emotion gaussians model for emotion-based music annotation and retrieval,” in Proceedings of the 20th ACM International Conference on Multimedia (Nara), 89–98. 10.1145/2393347.2396494

[B82] Waugh C. Shing E. Avery B. (2015). Temporal dynamics of emotional processing in the brain. Emot. Rev. 7, 323–329. 10.1177/1754073915590615

[B83] Widmann A. Schröger E. Wetzel N. (2018). Emotion lies in the eye of the listener: emotional arousal to novel sounds is reflected in the sympathetic contribution to the pupil dilation response and the P3. Biol. Psychol. 133, 10–17. 10.1016/j.biopsycho.2018.01.010 29378283

[B84] Xia G. Tay J. Dannenberg R. Veloso M. (2012). “Autonomous robot dancing driven by beats and emotions of music,” in Proceedings of the 11th International Conference on Autonomous Agents and Multiagent Systems (Richland, SC), 205–212.

[B85] Xianyu H. Li X. Chen W. Meng F. Tian J. Xu M.. (2016). “SVR based double-scale regression for dynamic emotion prediction in music,” in 2016 IEEE International Conference on Acoustics, Speech and Signal Processing (ICASSP) (Shanghai: IEEE), 549–553. 10.1109/ICASSP.2016.7471735

[B86] Yang Y.-H. Chen H. H. (2011). Prediction of the distribution of perceived music emotions using discrete samples. IEEE Trans. Audio Speech Lang. Process. 19, 2184–2196. 10.1109/TASL.2011.2118752

[B87] Yang Y.-H. Lin Y.-C. Cheng H.-T. Liao I.-B. Ho Y.-C. Chen H. H. (2008a). “Toward multi-modal music emotion classification,” in Pacific-Rim Conference on Multimedia (Tainan: Springer), 70–79. 10.1007/978-3-540-89796-5_8

[B88] Yang Y.-H. Lin Y.-C. Su Y.-F. Chen H. H. (2008b). A regression approach to music emotion recognition. IEEE Trans. Audio Speech Lang. Process. 16, 448–457. 10.1109/TASL.2007.91151334721556

[B89] Yang Y.-H. Liu C.-C. Chen H. H. (2006). “Music emotion classification: a fuzzy approach,” in Proceedings of the 14th ACM International Conference on Multimedia (New York, NY), 81–84. 10.1145/1180639.1180665

[B90] Zainab R. Majid M. (2021). Emotion recognition based on EEG signals in response to bilingual music tracks. Int. Arab J. Inf. Technol. 18, 286–296. 10.34028/iajit/18/3/4

[B91] Zhang H. Berg A. C. Maire M. Malik J. (2006). “SVM-KNN: discriminative nearest neighbor classification for visual category recognition,” in 2006 IEEE Computer Society Conference on Computer Vision and Pattern Recognition (CVPR'06) (New York, NY: IEEE), 2126–2136. 10.1109/CVPR.2006.301

[B92] Zhang Q. Chen X. Zhan Q. Yang T. Xia S. (2017). Respiration-based emotion recognition with deep learning. Comput. Indus. 92, 84–90. 10.1016/j.compind.2017.04.005

